# Modulation of the Plasma Kallikrein-Kinin System Proteins Performed by Heparan Sulfate Proteoglycans

**DOI:** 10.3389/fphys.2017.00481

**Published:** 2017-07-11

**Authors:** Guacyara Motta, Ivarne L. S. Tersariol

**Affiliations:** Departamento de Bioquímica, Escola Paulista de Medicina, Universidade Federal de São Paulo Sao Paulo, Brazil

**Keywords:** bradykinin, kininogen, kallikrein, proteoglycans, endocytosis, proteolysis, inflammation

## Abstract

Human plasma kallikrein-kinin system proteins are related to inflammation through bradykinin. In the proximity of its target cells, high molecular weight kininogen (H-kininogen) is the substrate of plasma kallikrein, which releases bradykinin from H-kininogen. Heparan sulfate proteoglycans (HSPGs) play a critical role in either recruiting kinin precursors from the plasma, or in the assembly of kallikrein-kinin system components on the cell surface. Furthermore, HSPGs mediate the endocytosis and activation of H-kininogen and plasma prekallikrein. In the presence of HSPGs (Chinese hamster ovary cell, CHO-K1, wild type cells) both heparin and heparan sulfate strongly inhibit the H-kininogen interaction with the cell membrane. H-kininogen is internalized in endosomal acidic vesicles in CHO-K1 but not in CHO-745 cells (mutant cells deficient in glycosaminoglycan biosynthesis). The endocytosis process is lipid raft-mediated and is dependent on caveolae. Both types of CHO cells do not internalize bradykinin-free H-kininogen. At pH 7.35, bradykinin is released from H-kininogen on the surface of CHO-745 cells only by serine proteases; however, in CHO-K1 cells either serine or cysteine proteases are found to be involved. The CHO-K1 cell lysate contains different kininogenases. Plasma prekallikrein endocytosis in CHO-K1 cells is independent of H-kininogen, and also prekallikrein is not internalized by CHO-745 cells. Plasma prekallikrein cleavage/activation is independent of glycosaminoglycans but plasma kallikrein formation is more specific on H-kininogen assembled on the cell surface through glycosaminoglycans. In this mini-review, the importance of HSPGs in the regulation of plasma kallikrein-kinin system proteins is shown.

## Plasma kallikrein-kinin system

In humans, the physiological plasma kallikrein-kinin system (KKS) pathway is composed of high molecular weight kininogen (H-kininogen), plasma pre (kallikrein) and bradykinin (BK). The KKS pathway is very important for inflammatory processes, through BK activities that are mediated by the constitutive G-protein-coupled receptor BK B2 (B2R) present in different cells (Regoli et al., 1996; Schmaier, 2016).

H-kininogen is a single polypeptide chain (120 kD), characterized as a six domain multifunctional protein playing antithrombotic, profibrinolytic, proinflammatory, andantimicrobial roles, and is an inhibitor of cysteine proteases and regulator of angiogenesis. After cleavage by plasma kallikrein (KAL) at Lys_362_-Arg_363_ and Arg_371_-Ser_372_, the H-kininogen turns BK-free H-kininogen into a two-chain protein composed of an N-terminal heavy chain (containing three domains, D1–D3, 62 kD) and a C-terminal light chain (containing two domains, D5–D6, 61 kD) bound to each other by a single disulfide bond between Cys_10_-Cys_596_ (Colman and Schmaier, 1997; Lalmanach et al., 2010).

The important peptide BK (Arg-Pro-Pro-Gly-Phe-Ser-Pro-Phe-Arg) was first described by Mauricio Rocha e Silva and co-workers (Rocha e Silva et al., 1949) as a substance that produces a slow delayed contraction of the isolated guinea pig ileum after incubation with either extracts of *Bothrops jararaca* venom or trypsin with the globulin fraction of dog plasma. BK corresponds to H-kininogen domain 4 (D4) and it reproduces many of the characteristics of an inflammatory state, such as changes in local blood pressure, edema, and pain, resulting in vasodilation and increased microvessel permeability (Bhoola et al., 1992). The release of pro-inflammatory and hyperalgesic mediators such as neuropeptides, leukotrienes, and cytokines, and the activation of sensory nerve terminals are the underlying causes of pro-inflammatory and nociceptive pharmacological effects induced by BK (Heitsch, 2003; Couture et al., 2014).

Plasma prekallikrein (PK) is the zymogen of KAL (EC 3.4.21.34), a serine protease synthesized predominantly in the liver. Its mRNA codes for a single chain protein which, according to the degree of glycosylation in the C-terminal portion, may appear as a doublet (85 and 88 kD) independent of the protein reduction. The classic activation of PK by factor XIIa occurs through cleavage of the Arg_371_-Ile_372_ bond producing KAL, containing an intact N-terminal heavy chain (53 kD) and a C-terminal light chain (33–36 kD) linked via a single disulfide bond between Cys_364_-Cys_484_. The protease domain is in the light chain and contains the catalytic domain (His_415_, Asp_464_, and Ser_559_) (Colman and Schmaier, 1997). PK and H-kininogen circulate as bimolecular complexes and ~75–80% of PK circulates as bound at plasma equilibrium conditions (Kaplan, 2014). The N-terminal heavy chain comprises four apple domains (A1–A4) and the binding affinity sites on PK for H-kininogen are A2>A4 ≈ A1>A3 (Renné et al., 1999). Two forms of active KAL have been described: α-KAL which presents both intact heavy and light chains, and β-KAL in which the heavy chain has undergone a single cleavage to produce fragments of ~18,000 and 28,000 kD, linked by disulfide bridges. Only one cleavage on the β-KAL heavy chain impairs the efficiency of BK release; therefore, the heavy chain does not seem to be necessary for non-specific cleavages of H-kininogen by β-KAL (Motta et al., 1989; Page and Colman, 1991).

KAL plays a central role in a variety of proteolytic systems, such as the intrinsic pathway of coagulation, the KKS, the fibrinolysis pathway, the renin–angiotensin system, and the complement pathways. Therefore, it can be considered an important regulator in the pathogenesis of thrombosis, inflammation, and blood pressure (Kolte and Shariat-Madar, 2016).

## Plasma kallikrein-kinin system and cell surface interaction

In the intravascular compartment, the interaction and activation of KKS on the cell surface of platelets, neutrophils, endothelial cells, and macrophages have been described (Colman, 2006; Schmaier and McCrae, 2007; Barbasz et al., 2008). The membrane-binding proteins of H-kininogen or BK-free H-kininogen on endothelial cells include the globular domains of complement factor C1q receptor (gC1qR), urokinase plasminogen activator receptor (uPAR) and cytokeratin 1 (CK1), in which the affinity measured using surface plasmon resonance is gC1qR>CK1>soluble uPAR, indicating that gC1qR is dominant for binding (Pixley et al., 2011).

Once assembled on the cell surface H-kininogen functions as a putative receptor for PK; therefore, a lower affinity of PK can be detected in the absence of added H-kininogen; the cell-associated PK is rapidly converted to KAL in the presence of H-kininogen, that is proteolyzed during the KAL formation producing BK, and Motta et al. (1998) first describe the factor XII independent pathway for contact factor activation on human umbilical vein endothelial cells (HUVECs) that regulate BK production.

BK plays an important role in regulating angiogenesis through upregulation of endogenous basic fibroblast growth factor through the inductive G-protein-coupled receptor BK B1 or viral fibroblast growth factor through the B2R, by regulation of vascular permeability or stimulation of cell proliferation through the B2R. The H-kininogen cleavage by KAL promotes conformational changes in BK-free H-kininogen and exposure of domain 5 (D5), which inhibits endothelial cell migration and proliferation, both of which play a role in angiogenesis (Guo and Colman, 2005). This potent anti-angiogenic activity occurs through tight-binding to cell surface tropomyosin which also induces endothelial cell apoptosis (McCrae et al., 2005). The peptide sequence on H-kininogen D5, comprising the residues His_497_–K_520_, is important for homing of both T and B cells to lymph nodes, which is a powerful mechanism of coupling inflammation to adaptive immunity (Ponda and Breslow, 2016).

PK is brought to the endothelial surface by H-kininogen or BK-free H-kininogen since it binds to a peptide sequence (Trp_569_-Lys_595_) on domain 6 (D6) (Vogel et al., 1990). The activation of the KKS on endothelial cells needs both H-kininogen and zinc ions (Røjkjaer et al., 1998). Zinc ions mediate both H-kininogen and BK-free H-kininogen binding with high affinity to endothelial cells (Reddigari et al., 1993) and through domain 5 (D5) both forms also bind with high affinity to heparin, a negatively charged glycosaminoglycan (GAG; Björk et al., 1989).

Heparin interacts with Lys and/or Arg residues within proteins via electrostatic interaction and zinc facilitates binding to heparin by binding to expose His residues and stabilizes the non-covalent complex between heparin and both forms of H-kininogen (Lin et al., 2000). H-kininogen D5 contains two subdomains, a His-Gly-rich region (Lys_420_-Asp_474_) and a His-Gly-Lys-rich region (His_475_-Lys_502_); H-kininogen exhibits a higher affinity to heparin (lower *K*_*D*_) than BK-free H-kininogen because the former loses a peptide sequence (Ser_372_-Arg_419_) which corresponds to a third cleavage by KAL after BK release from H-kininogen (Nakayasu and Nagasawa, 1979).

Several proteinases from two major classes, the serine and cysteine proteases, cleave H-kininogen; nevertheless, they differ in specificity and not all of them release BK or Lys-BK. Among the serine proteases are KAL, tissue kallikrein, trypsin, factor XIIa, factor XIa, plasmin and neutrophil elastase (Mauron et al., 2000). At inflammatory sites, a combined action of tryptase/neutrophil elastase or KAL/neutrophil elastase may release kinins. Among cysteine proteases are calpains and cathepsin L that release kinins from H-kininogen at slightly acidic pH, suggesting these enzymes as candidates for kinin production in a pathophysiological environment (Lalmanach et al., 2010).

The findings of the current research group show that cathepsin B has kininogenase activity at pH 6.3, which is improved in the absence of divalent cations (Zn^2+^, Mg^2+^, and Ca^2+^), however, at pH 7.35 cathepsin kininogenase activity is impaired suggesting that H-kininogen is a substrate for cathepsin B under pathophysiological conditions (Barros et al., 2004a).

## Glycosaminoglycans and inflammation

Heparan sulfate (HS) polysaccharide chains are ubiquitous GAGs in animal cells (Dietrich et al., 1983). These classes of molecules are heteropolysaccharides composed of repeating units of disaccharides, a uronic acid residue, either D-glucuronic acid or L-iduronic acid, and D-glucosamine with *N*- and 6-*O*-sulfates and *N*-acetyl substitutions (Dietrich et al., 1998). HS occurs at the cell surface and in the extracellular matrix (ECM) as proteoglycans (PGs). Most of the cellular HS derives from the syndecans and glypicans PGs. The syndecan family is associated with the cell membranes via transmembrane core proteins (Yanagishita and Hascall, 1992; Elenius and Jalkanen, 1994), and the glypican family is anchored by glycosilyl phosphatidylinositol-anchor core proteins (David, 1993). In addition, heparan sulfate proteoglycans (HSPGs) are present in basement membranes as the perlecan family (Iozzo et al., 1994).

HSPGs regulate a wide variety of biological process including hemostasis, inflammation, angiogenesis, growth factors, and cell adhesion, and play a major role in the ECM (Conrad, 1998). The interactions occurring in the ECM with HSPG have shown the direct regulation of the fibroblast growth factors (FGFs) diffusion that can determine the shape of growth concentration gradients in development, as well as the storage and release of FGFs in tissue homeostasis. The growth factor/morphogen-type signals generated by FGFs require the assembly of the ternary complex of FGF ligand, FGF receptor (FGFR) and HS, which engages both the ligand and receptor, acting as a co-receptor (Li et al., 2016). The heparin-related GAG chains of the HSPGs are involved in the inflammatory process through binding and modification of the activity of several molecules (Götte, 2003).

Heparin and HSPGs can alter the proteolytic activity of various serine proteases of the coagulation cascade as well as their natural inhibitors, the serpins (Ermolieff et al., 1994; Gettins et al., 1996; Fath et al., 1998). HSPGs can also control the activity of several serine proteases directly involved in the inflammatory response. Data from the literature show that ectodomains of syndecan-1 and syndecan-4 present in acute inflammatory fluid are able to regulate the proteolytic activity of cathepsin G and elastase. The enzymatic degradation of polysaccharide chains of HS in HSPGs promotes strong inactivation of elastase and cathepsin G serine proteinases present in the inflammatory fluid (Kainulainen et al., 1998). The syndecan-1 ectodomain protects the inhibition of cathepsin G by the natural inhibitors, the serpin α1-antichymotrypsin and squamous cell carcinoma antigen-2 and protects the inhibition of elastase by α1-antichymotrypsin.

At the neutrophil surface HSPGs bind human neutrophil elastase (HNE) and preserve its activity by protecting it from inhibition by α1-antitrypsin and secretory leukocyte peptidase inhibitor and focus the activity of HNE to the pericellular environment (Campbell and Owen, 2007). Syndecan knockout mice show deficits in tissue repair (Bishop et al., 2007). In mast cells, intracellular heparin PG serglycin compartmentalizes various proteolytic enzymes, among them chymase and tryptase proteases (Pejler et al., 2007). The major intracellular PG of hematopoietic cells is serglycin PG which has been related to sorting and packing of granule HNE (Niemann et al., 2007).

HNE can also regulate tissue inhibitor of metalloproteases-1 (TIMP-1) and metalloproteinase-9 (MMP-9) activities. In the pro-MMP-9/TIMP-1 complex, HNE preferentially inactivates TIMP-1 and renders pro-MMP-9 activity through MMP-3 (Itoh and Nagase, 1995). TIMP-1, as an inhibitor of MMP-2 and MMP-9, is tightly correlated to the maintenance of the ECM structure (Murphy and Nagase, 2008). An MMP-9/TIMP-1 imbalance, caused by the proteolytic activity of HNE, has an important pathophysiological role in the sputum of patients with cystic fibrosis (Jackson et al., 2010), intracranial hemorrhage (Alvarez-Sabín et al., 2004), abdominal aortic aneurysm (Wiernicki et al., 2010) and bone resorption (Bord et al., 1997).

Heparin increases eight-fold the initial rate of pro-MMP-9 autolytic activation (Crabbe et al., 1993). Interestingly, the major clinical complication of heparin anticoagulant therapy is hemorrhage (Zidane et al., 2000) and osteoporosis by increasing bone resorption (Rajgopal et al., 2008) as a side effect. Heparin accelerates the rate of hydrolysis of TIMP-1 by HNE and the most important adverse effects of heparin therapy are related to excessive activation of MMP-9 (Nunes et al., 2011).

Human neutrophil proteases can release Met-Lys-BK and BK from low-(L) and H-kininogens (Stuardo et al., 2004). The cooperative action of mast cell tryptase and HNE can release BK from H-kininogen and L-kininogen, with efficiency not much lower than that of KAL. Interestingly, the release of BK from oxidized kininogen substrate by the tryptase/elastase mixture was not altered (Kozik et al., 1998). Therefore, the plasma level of BK is higher in wild-type C57BL/6 mice than in elastase-deficient mice (Sahoo et al., 2014). It is important to note that both tryptase (Hallgren et al., 2005; Pejler et al., 2007) and neutrophil elastase proteases (Campbell and Owen, 2007; Niemann et al., 2007) have their proteolytic activity controlled by heparin and HSPGs.

## Heparan sulfate proteoglycans and the plasma kallikrein-kinin system

In the early 2000s, not only HS but also chondroitin sulfate (CS) GAGs were indicated as putative receptors that accumulate H-kininogen on the cell surface of HUVECs and other cells in a zinc-dependent manner, along with H-kininogen binding proteins, the integrin receptor Mac-1/α_M_β_2_, p33/gC1qR, uPAR, CK1, thrombospondin-1 and glycoprotein-Ib (Renné et al., 2000; Renné and Müller-Esterl, 2001; Fernando et al., 2003).

Previous studies concerning KKS purified protein assembly on the ECM of different endothelial cell lines, from rabbit aorta endothelial cells (RAECs) and ECV304 cells, suggest that not only the cell membranes but also the ECM produced by these cells assemble H-kininogen/PK as a complex and form KAL that activates pro-urokinase, which in turn activates plasminogen (Motta et al., 2001).

On the RAEC surface or ECM, GAGs also influence this process because in the presence of Zn^2+^ heparin abolishes H-kininogen binding which is reduced by HS and other GAGs; in contrast, only heparin reduces H-kininogen binding to the ECV304 surface or ECM. Heparin does not modify PK binding and activation in the presence of H-kininogen assembled to the cell surface or ECM. In the fluid phase, heparin in the presence of Zn^2+^augments the BK release from H-kininogen after hydrolysis by KAL. GAGs that accumulate in inflammatory fluids or are used as a therapeutic drug (e.g., heparin) could act as pro- or anti-inflammatory mediators depending on different factors within the cell environment (Gozzo et al., 2011).

**Figure 1:**
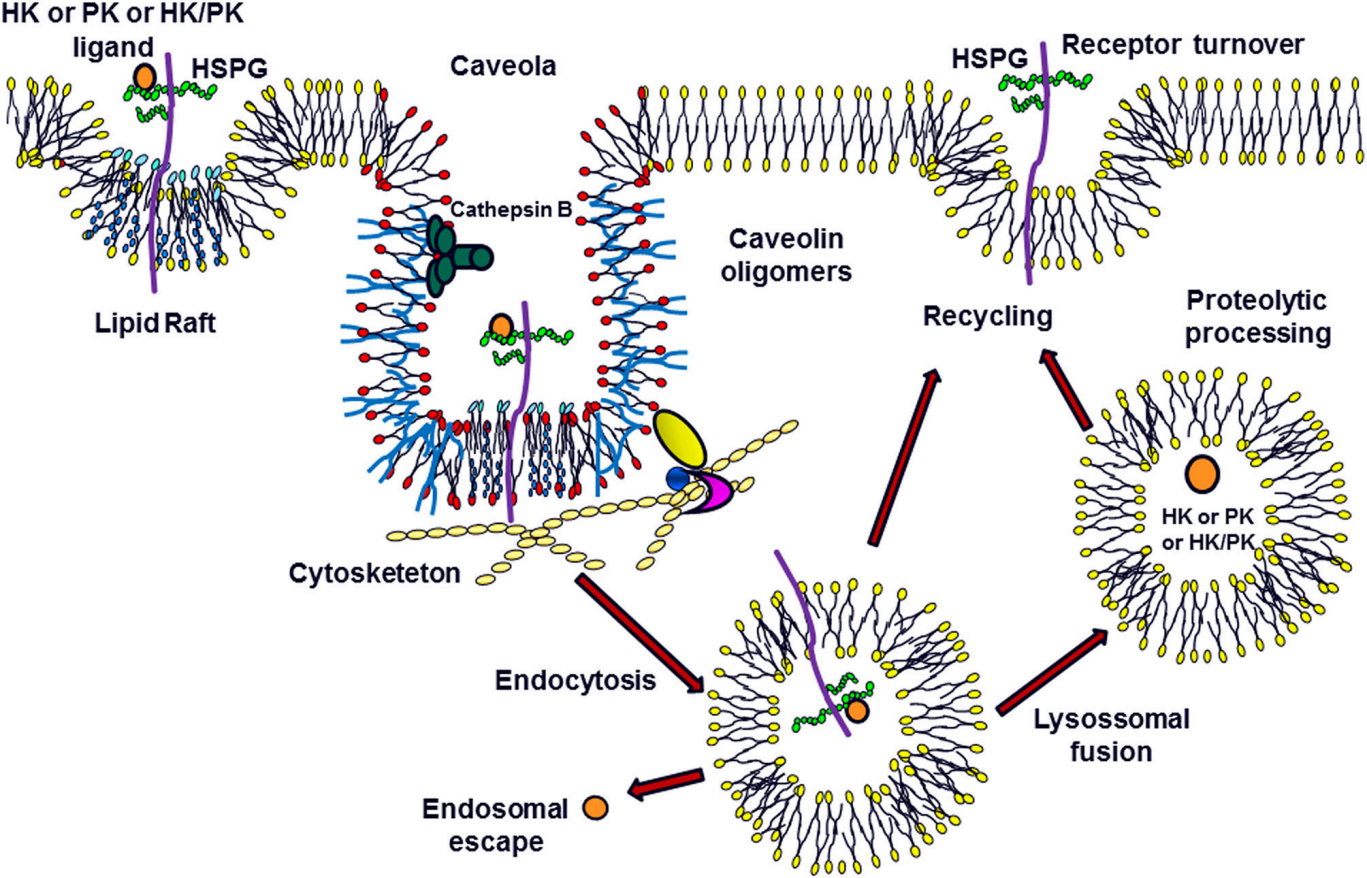
Modulation of Plasma Kallikrein-Kinin System: the Role of Heparan Sulfate Proteoglycans. H-kininogen (HK) or plasma prekallikrein (PK) alone, or as a complex (HK/PK), are associated with the cell surface. The heparan sulfate proteoglycans (HSPG) in the membrane microdomains can function as ligands for these molecules, promoting caveolae-mediated endocytosis. Once present in the primary endosome, these ligands can escape to the cytoplasm or be processed after the fusion between endosomes and lysosomes. Furthermore, these molecules may undergo proteolysis on the cell surface by serine or cysteine proteases. The HSPGs can be recycled to the cell surface.

The current research group is the first to show that the interaction of H-kininogen and PK on the cell surface mediated by HSPGs results in endocytosis (Melo et al., 2009; Veronez et al., 2014; Damasceno et al., 2015; Figure 1). H-kininogen interacts with cellular sites in either RAECs or epithelial CHO cells, wild type CHO-K1 cells and CHO-745 cells, mutant cells deficient in xylosyltransferase and as consequence is involved in GAG biosynthesis. The HS chain of HSPG is involved in H-kininogen binding to RAECs and CHO-K1 cells. The interaction parameters of H-kininogen with RAECs and CHO cells are strongly dependent on temperature and the interaction between H-kininogen and cells was not totally reversible at 37°C; the interaction with CHO cells is related to vital processes also influenced by ATP synthesis; the H-kininogen interaction in CHO cells is independent of cycloheximide, indicating that new protein synthesis does not influence H-kininogen binding. GAGs are not the main cellular receptors; however, GAGs modulate the cellular receptors of H-kininogen in CHO cells (Melo et al., 2009).

In CHO-K1 cells, after H-kininogen interacts with HS at the cell surface, it is internalized by endocytosis followed by a fusion step within an acidic endosomal compartment. The endocytosis process of H-kininogenin CHO-K1 cells is disrupted by pretreatment of the cells using chloroquine, an alkalinizing agent of acid endosomes; cholesterol depletion by methyl-β-cyclodextrin also inhibits endocytosis; the endocytosis of H-kininogen is independent of transferrin endocytosis, strongly indicating that the endocytosis of H-kininogen is not dependent on clathrin. The membrane lipid raft domains/caveolae mediate the endocytotic process of H-kininogen, as the colocalization of H-kininogen with caveolin-1 has been demonstrated (Melo et al., 2009; Damasceno et al., 2015).

In contrast, CHO-745 cells, which are almost completely devoid of GAGs, do not take up intact H-kininogen; the inhibition of GAG sulfation blocks the endocytosis process, and the integrity of H-kininogen is very important for internalization since BK-free H-kininogen does not become internalized (Veronez et al., 2014; Damasceno et al., 2015).

The cysteine proteases play a role in H-kininogen processing on the cell surface and in acidic endosomal vesicles. At neutral pH and after H-kininogen assembly on the cell surface, kinin generation was much higher in CHO-K1 cells, and tumorigenic cells, when compared with RAECs, as expected, as either serine or cysteine proteases are involved in kinin release. In CHO-745 cells, only serine protease activity is detected consistently with the regulation of cysteine proteases by GAGs/PGs in H-kininogen processing and kinin release (Melo et al., 2009; Damasceno et al., 2015). The lysate fraction of CHO-K1 cells possesses kininogenase activity at pH7.4 but the total cleavage of intact H-kininogen is more effective at pH 5.5 after a 4 h incubation. In crude lysates, the hydrolysis patterns are quite similar suggesting a kininogenase activity at a pH optimum of 5.5 that can still work at pH 7.4, albeit more slowly. The antipain-Sepharose affinity chromatography of the CHO-K1 cell lysate fraction successfully separates different kininogenases (Damasceno et al., 2015).

Other groups have shown that H-kininogen binding to HS and CS efficiently interferes with BK release by KAL, in plasma and on endothelial surfaces (Renné et al., 2005), and two enzymes purified from HUVECs are described as PK activators on cells, the heat shock protein 90 and prolylcarboxypeptidase (Bryant and Shariat-Madar, 2009).

PK interacts directly with the endothelial cell surface independent of the presence of H-kininogen (Motta et al., 1998). The PK interaction with CHO and ECV304 cells shows that although the process is temperature-dependent, it also depends on the GAG composition of the cell type. HSPGs direct PK to acidic endosomal vesicles and the endocytosis process of PK/KAL depends on GAGs, but not those bound to H-kininogen. PK interaction with GAGs does not disturb its cleavage/activation; therefore, H-kininogen bound to GAGs may modulate PK as a control mechanism of KAL activity (Veronez et al., 2014). Either cathepsin B or cathepsin L, which are lysosomal cysteine proteases, might hydrolyze PK or KAL (Barros et al., 2004b).

Figure 1 illustrates the concept that after blood vessel injury, H-kininogen and PK or KAL can interact and bind to the HSPGs of non-endothelial cells. On lipid raft domains/caveolae or endosomes, HSPGs mediate the endocytosis of intact H-kininogen, which can be processed by different classes of proteases. The cleavage produces H-kininogen fragments that may play roles as cystatins, effectors of innate immunity, inflammation, angiogenesis, and coagulation. The KAL proteolytic balance of H-kininogen/BK free H-kininogen forms is controlled by GAGs, which may promote this control in two different ways: controlling the proteolytic activity of the ternary complex GAG/H-kininogen/KAL or the cellular compartmentalization of KKS proteins via endocytosis.

## Author contributions

GM and IT conceived, designed and wrote the review. GM has experience in plasma kallikrein/kinin system and IT has experience in proteoglycans field. Both authors are professors at Department of Biochemistry at Medical School (Escola Paulista de Medicina) which has very important tradition and contribution in both fields. Both authors work also on cell biology and enzymes.

### Conflict of interest statement

The authors declare that the research was conducted in the absence of any commercial or financial relationships that could be construed as a potential conflict of interest. The reviewer CO and handling Editor declared their shared affiliation, and the handling Editor states that the process nevertheless met the standards of a fair and objective review.

## Abbreviations

BK: bradykinin
CHO: cell line from Chinese hamster ovary cells
CK1: cytokeratin 1
CS: chondroitin sulfate
ECM: extracellular matrix
ECV304: cell line from spontaneously transformed human umbilical vein endothelial cells
gC1qR: globular domains of complement factor C1q receptor
GAG: glycosaminoglycan
B2R: G-protein-coupled receptor bradykinin B2
H-kininogen: high molecular weight kininogen
HNE: human neutrophil elastase
HUVECs: human umbilical vein endothelial cells
HS: heparan sulfate
HSPGs: heparan sulfate proteoglycans
KKS: plasma kallikrein-kinin system
KAL: plasma kallikrein
MMP-9: metalloproteinase-9
PGs: proteoglycans
PK: plasma prekallikrein
RAECs: rabbit aorta endothelial cell line
TIMP-1: tissue inhibitor of metalloproteases-1.
